# Diel activity patterns of vector mosquito species in the urban environment: Implications for vector control strategies

**DOI:** 10.1371/journal.pntd.0011074

**Published:** 2023-01-26

**Authors:** André B. B. Wilke, Adequate Mhlanga, Allisandra G. Kummer, Chalmers Vasquez, Maday Moreno, William D. Petrie, Art Rodriguez, Christopher Vitek, Gabriel L. Hamer, John-Paul Mutebi, Marco Ajelli

**Affiliations:** 1 Laboratory for Computational Epidemiology and Public Health, Department of Epidemiology and Biostatistics, Indiana University School of Public Health, Bloomington, Indiana, United States of America; 2 Miami-Dade County Mosquito Control Division, Miami, Florida, United States of America; 3 Public Health Department, City of Brownsville, Brownsville, Texas, United States of America; 4 Center for Vector-Borne Diseases, The University of Texas Rio Grande Valley, Texas, United States of America; 5 Department of Entomology, Texas A&M University, College Station, Texas, United States of America; 6 Arboviral Diseases Branch (ADB), Division of Vector-Borne Diseases (DVBD), Centers for Disease Control and Prevention (CDC), Fort Collins, Colorado, United States of America; QIMR Berghofer Medical Research Institute, AUSTRALIA

## Abstract

Mathematical models have been widely used to study the population dynamics of mosquitoes as well as to test and validate the effectiveness of arbovirus outbreak responses and mosquito control strategies. The objective of this study is to assess the diel activity of mosquitoes in Miami-Dade, Florida, and Brownsville, Texas, the most affected areas during the Zika outbreak in 2016–2017, and to evaluate the effectiveness of simulated adulticide treatments on local mosquito populations. To assess variations in the diel activity patterns, mosquitoes were collected hourly for 96 hours once a month from May through November 2019 in Miami-Dade County, Florida, and Brownsville, Texas. We then performed a PERMANOVA followed by a SIMPER analysis to assess whether the abundance and species richness significantly varies at different hours of the day. Finally, we used a mathematical model to simulate the population dynamics of 5 mosquito vector species and evaluate the effectiveness of the simulated adulticide applications. A total of 14,502 mosquitoes comprising 17 species were collected in Brownsville and 10,948 mosquitoes comprising 19 species were collected in Miami-Dade County. *Aedes aegypti* was the most common mosquito species collected every hour in both cities and peaking in abundance in the morning and the evening. Our modeling results indicate that the effectiveness of adulticide applications varied greatly depending on the hour of the treatment. In both study locations, 9 PM was the best time for adulticide applications targeting all mosquito vector species; mornings/afternoons (9 AM– 5 PM) yielded low effectiveness, especially for *Culex* species, while at night (12 AM– 6 AM) the effectiveness was particularly low for *Aedes* species. Our results indicate that the timing of adulticide spraying interventions should be carefully considered by local authorities based on the ecology of the target mosquito species in the focus area.

## Author summary

Mathematical models have been widely used to study vector mosquitoes as well as to test the effectiveness of arbovirus outbreak response and mosquito control strategies. However, due to the lack of empirical data, there are no studies focusing on the effectiveness of adulticide applications at different hours of the day to control different mosquito populations. This study leveraged a unique dataset in which approximately 25,000 mosquitoes comprising 19 species were collected hourly in Miami-Dade County, Florida, and Brownsville, Texas. We then developed a mathematical model to simulate the population dynamics of five mosquito vector species to evaluate the effectiveness of adulticide spraying at different times of the day and at different frequencies. Mosquito community composition and abundance varied significantly throughout the day in both Brownsville and Miami-Dade County with more than 10-fold differences during the day. Depending on the target vector species, the application of adulticides at a given hour of the day may lead to drastically different results, although we found some common patterns such as the remarkable effectiveness of interventions performed at 9 PM.

## Introduction

The invasion and proliferation of mosquito vector species across new areas directly affect the epidemiology of mosquito-borne diseases, especially in complex socio-ecological urban ecosystems [1–3]. Due to the increase in the presence and abundance of vector mosquito species, major cities in the contiguous United States have been affected by local arbovirus transmission [4,5]. In particular, southern Florida and southern Texas have historically been afflicted by arbovirus outbreaks. They were the most affected areas of the United States during the Zika virus outbreak of 2016 [3,6] and have remained the theater of locally transmitted arboviral infections ever since [5,7,8].

The constant increase in the number of arboviral infections in historically unaffected areas is most likely due to the increase in the contact rate between mosquito vector species and human hosts [9,10]. Controlling mosquitoes is considered the most effective way to prevent arbovirus outbreaks; however, controlling mosquitoes in urban areas is a difficult task [11]. Large mosquito populations, including vector species, are now harbored in urban areas thanks to an overabundance of resources (including widely available artificial aquatic habitats for larval development and human hosts for blood-feeding), lack of or reduced density of natural predators, and reduced larval competition [12–14].

One of the most common strategies to control mosquitoes in urban areas is to reduce the number of active host-seeking females by spraying adulticides [15], especially in non-emergency situations, and in developing countries with limited access to more expensive and ecologically friendly alternatives such as bacterial larvicides and insect growth regulators. One of the most important elements that separate successful from unsuccessful adulticide spraying is the extent to which the target adult mosquito population is exposed to the adulticide [16]. To effectively reduce a mosquito population, adulticide applications must coincide with the period when the mosquitoes are most active (actively flying). On the other hand, adulticide interventions will have limited effects on resting adult mosquitoes. Therefore, knowing the period(s) of the day mosquitoes are most active is of paramount importance for vector control operations based on adulticide treatments and for the prevention and control of local arboviral transmission. To this aim, we collected mosquitoes hourly for 96 hours once a month for seven months in Miami-Dade County, Florida, and Brownsville, Texas. We then developed a mathematical model informed with the collected data to simulate “in silico” the population dynamics of 5 mosquito vector species and the potential effect of adulticide applications at different times of the day and frequencies.

## Methods

### Mosquito collection and study sites

Mosquitoes were collected by BG-Sentinel 2 traps (Biogents AG, Regensburg, Germany) baited with BG Lures and dry ice as a source of carbon dioxide in Miami-Dade County, Florida, and Brownsville, Texas, as described in Mutebi et al. [17]. The study locations were chosen due to their history of arboviral outbreaks, including West Nile, yellow fever, dengue, and Zika viruses. In each city, four collection sites were selected to capture the natural variation in the diel activity patterns of mosquito species due to their inherent socio-ecological features. BG-Sentinel 2 traps are the gold standard to assess the community composition of mosquitoes in urban environments. They mimic a potential host by using BG-Lures that release attractants consisting of carbon dioxide, lactic acid, ammonia, and caproic acid, substances that are found on animal skin thus attracting female mosquitoes seeking blood sources. In addition, the BG traps were baited with dry ice to release carbon dioxide, which has been shown to significantly increase the capture of diverse mosquito species [18]. Male mosquitoes collected during this study were considered accidental catches and, since they were not consistently collected, they were not considered informative and were not included in the analyses.

Mosquito collections were made from May 2019 to November 2019 as previously described by Mutebi et. al. [17]. Mosquitoes were collected during the warmer months of the year (at the study sites) to maximize the number of collectable specimens. Each BG-Sentinel trap was monitored every hour for 96 hours at each location once every month from May to November 2019. Every hour, the BG-Sentinel traps were serviced by project personnel, and the collection bags were removed from the traps and replaced with empty ones. The bags of mosquitoes were then labeled and transported to the laboratory under refrigeration, where they were subsequently identified to species on chill tables by using entomological keys [19].

### Diversity analysis

To determine the extent to which the diel activity patterns of each species as well as the abundance and species richness varied in each hour of the day, and if these differences were statistically significant, we assessed the hourly community composition, richness, and abundance of mosquitoes. To assess variations in the diel activity patterns of mosquito species in each city, we organized the data into six groups (group 1 = 12 AM to 3 AM; group 2 = 4 AM to 7 AM; group 3 = 8 AM to 11 AM; group 4 = 12 PM to 3 PM; group 5 = 4 PM to 7 PM; and group 6 = 8 PM to 11 PM) and performed a PERMANOVA with 9,999 permutations based on Bray-Curtis (abundance-based) and the Jaccard (incidence-based) indices [20]. Then, we used the SIMPER method for assessing which species has contributed the most to the observed differences between groups of samples [21]. To assess hourly variations in the mosquito community composition, we used the Dominance (1-Simpson) index, in which values closer to 0 indicate species are equally present and values closer to 1 indicate the presence of highly dominant species. Analyses were done using PAST v4 [22].

### Stochastic compartmental model

We used a stochastic compartmental model to simulate the population dynamics of mosquito species to determine the effectiveness of adulticide applications at different times of the day. We selected the most epidemiologically important mosquito vector species that were collected in Brownsville, Texas: *Aedes aegypti*, *Aedes albopictus*, *Culex coronator*, *Culex nigripalpus*, and *Culex quinquefasciatus*; and in Miami-Dade County, Florida: *Ae*. *aegypti*, *Cx*. *coronator*, *Cx*. *nigripalpus*, and *Cx*. *quinquefasciatus* [23–29].

The mathematical model for the dynamics of the vector mosquito populations considers four developmental stages: eggs (E), larvae (L), pupae (P), and the adult female (A) population.

In our mathematical model, we assume that under suitable conditions, eggs hatch into larvae at rate d_E_ and some suffer natural mortality at rate m_E_. Upon feeding on micro-organisms, and after moulting three times, the larvae then develop into pupae at rate d_L_. In our model, we consider the carrying capacity K of the larval habitats to limit the maximum number of eggs that can be allowed to develop. As such, we assume no further competition for food among the larvae; hence they only suffer natural mortality at rate m_L_. Pupae develop to become adult mosquitoes at rate d_P_ and die at rate m_P_. We considered a 1:1 sex ratio among adult mosquitoes [30]. In the adult stage, we consider only female adult mosquitoes as males are not epidemiologically relevant. We considered the gonotrophic cycles to occur at rate d_A_ and n_E_ represents the average numbers of eggs laid per each oviposition. Finally, adult mosquitoes die at rate m_A_. All model parameters are species-dependent.

The described process can mathematically be represented by the following system of equations:

$$\dot{E}=n_{E}d_{A}\left(1-\frac{E}{K}\right)A-m_{E}E-d_{E}E$$

$$\dot{L}=d_{E}E-m_{L}L-d_{L}L$$

$$\dot{P}=d_{L}L-m_{P}P-d_{P}P$$

$$\dot{A}=\frac{1}{2}d_{P}P-m_{A}A$$

In our mathematical model, transitioning from one life stage to another is a stochastic process modelled as binomial transitions. We utilize a discrete-time stochastic version of our mathematical model, with time a step of 0.1 days. The model was implemented in R.

### Development and mortality rates

All the development and mortality rates used in our model are temperature- and species-dependent and we used a constant temperature of 25 degrees Celsius. The use of a constant temperature guarantees that the model can reach a stable equilibrium for each species, which makes our analysis independent from the initial condition. The development and mortality rates for *Ae*. *albopictus* were obtained from Delatte et al. [30] and implemented as in Poletti et al. [31]. The larvae and pupae development ratios for *Ae*. *aegypti* and *Cx*. *quinquefasciatus* were obtained from Grech et al. [32]. The egg hatching and the gonotrophic times for the *Ae*. *aegypti* were obtained from Farnesi et al. [33] and Garcia-Rejon et al. [34] respectively. Additionally, the egg hatching and the gonotrophic cycle for the *Cx*. *quinquefasciatus* were taken from Handel [35] and Laporta & Sallum [36] respectively. The mortality rates for eggs, larvae, pupae, and adult *Cx*. *quinquefasciatus* mosquitoes were obtained from Ewing et al. [37] and Watanabe et al. [38], and Farnesi et al. [33], Yang et al. [39] and Yang et al. [40] for *Ae*. *aegypti*, respectively. Model parameters are reported in S5 Table.

### Simulation model of adulticide spraying

Four frequencies of applications of adulticides at a given time of the day are considered: i) twice a day (within the same hour, resembling real-world mosquito control operations) for 5 consecutive days, ii) once a month for two months, iii) once a week for two months, and iv) once every other week for two months. We consider adulticide spraying to affect only adult mosquitoes that are active at the time of spraying. As such, for each study location, hour of the day, and mosquito species, we estimated the fraction of active mosquitoes as: ${r_{ij}(t)=C}_{ij}(t)/\underset{j}{\max}\left[C_{ij}(t)\right],$ where *C*_*ij*_(*t*) represents the estimated number of active adult mosquitoes in location *i* = {*Brownsville*, *Miami*} of species *j*, at time of the day *t*. To account for the uncertainty in the number of captured mosquitoes at each time of the day, *C*_*ij*_(*t*) has been sampled from a Poisson distribution of mean ${\tilde{C}}_{ij}(t)$, representing the observed number of captured mosquitos in location *i*, of species *j*, at time of the day *t*. Upon spraying, the adult mosquito population is instantly reduced by *σr*_*ij*_(*t*), where *σ* represents the spraying efficacy. Spraying efficacy is set at 50% in the main analysis, while 30% and 70% are investigated in the supplementary analyses. To estimate the uncertainty in the effectiveness of the interventions, for each location, mosquito vector species, and time of the adulticide spraying, we performed 1,000 simulations, one for each sampled value of *C*_*ij*_(*t*). To estimate the effect of the application of adulticides, we run the model until equilibrium is reached and only then we simulate interventions.

## Results

### Descriptive analysis

A total of 14,502 mosquitoes comprising 17 species were collected in Brownsville and 10,948 mosquitoes comprising 19 species were collected in Miami-Dade County. *Aedes aegypti* was the most common mosquito species collected every hour of the day in both Brownsville and Miami-Dade County. It was also the most abundant species, totaling 7,024 and 4,444 collected specimens in Brownsville and Miami-Dade County, respectively. *Culex quinquefasciatus* was the second most abundant species in Brownsville with 4,473 specimens; however, it was only collected from 6 PM to 7 AM. On the other hand, *Cx*. *quinquefasciatus* was not abundant in Miami-Dade County, it was the fifth most abundant species collected (237 specimens), mostly between dusk and dawn (S1 and S2 Tables).

### Species richness

**Fig 1:**
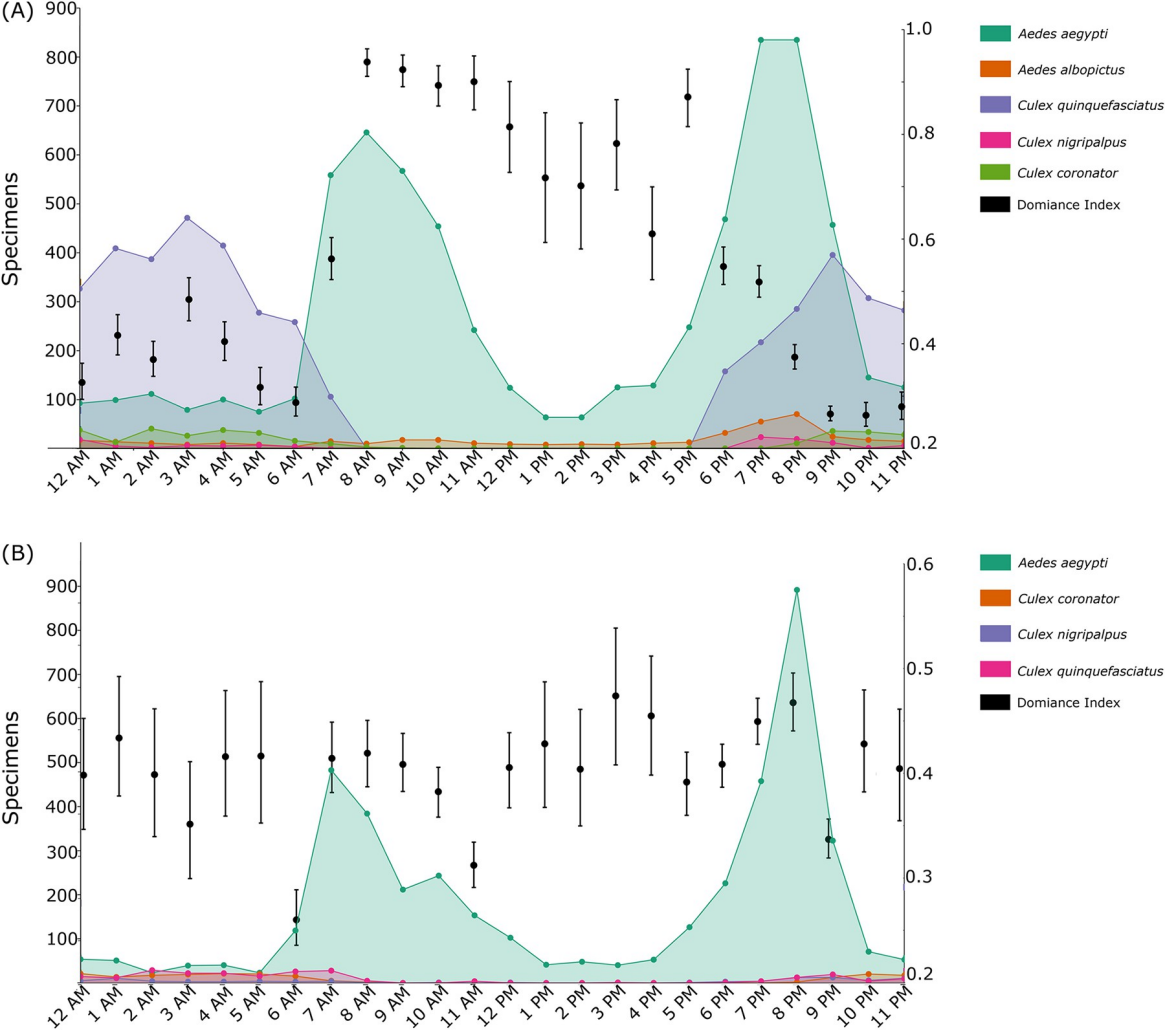
Diel activity patterns of mosquito vector populations. **(A)** Total number of female adult mosquito specimens collected over the entire study period by hour of collection in Brownsville, Texas. The figure reports vector mosquito species only; S1 Fig shows all the collected species. The Dominance Index is shown in black (mean and 95% CI). **(B)** Same as A, but for Miami-Dade County, Florida.

Species richness varied greatly according to the time of the day with most species being more common between dusk and dawn. In Brownsville, the highest number of species was 15 at 10 PM and the lowest was 3 at 11 AM, 1 PM, and 2 PM. In Miami-Dade County, the highest number of species collected was 14 at 9 PM and the lowest was 4 species at 9 AM (Figs 1 and S1).

The PERMANOVA analyses using the Bray-Curtis and Jaccard indices yielded significant results for the diel activity patterns of mosquito species in both Brownsville and Miami-Dade County. The subsequent SIMPER analyses resulted in distinct scenarios. In Brownsville, *Psorophora cyanescens*, *Culex erraticus*, *Aedes sollicitans*, and *Psorophora ciliata* contributed approximately 75% of all the variation in the mosquito community composition. Important mosquito vector species such as *Cx*. *nigripalpus* (7.6%), *Cx*. *quinquefasciatus* (<0.001%), *Ae*. *aegypti* (<0.001%), and *Cx*. *coronator* (<0.001%) did not contribute substantially to the variation (Table 1). A different scenario was observed in Miami-Dade County, with *Ae*. *aegypti*, *Aedes taeniorhynchus*, and *Wyeomyia vanduzeei* accounting for more than 80% of all the variation in the mosquito community composition. Vector species such as *Ae*. *albopictus*, *Cx*. *quinquefasciatus*, *Cx*. *coronator*, and *Cx*. *nigripalpus* did not contribute substantially to the variation in the community composition (S3 and S4 Tables).

**Table 1:** Permutational Multivariate Analysis of Variance (PERMANOVA) and SIMPER (Similarity Percentage) analysis of which species contributed the most to the observed differences in Brownsville, Texas, and Miami-Dade County, Florida.

	Brownsville		Miami-Dade	
PERMANOVA	Bray-Curtis	Jaccard	Bray-Curtis	Jaccard
Permutations	9999	9999	9999	9999
Total sum of squares	2.644	3.313	4.78E+06	2.739
Within-group sum of squares	1.024	1.341	2.20E+06	1.119
F	5.7	5.293	4.212	5.213
*P*	0.0004	0.0001	0.0018	0.0001
**SIMPER**	Average dissimilarity	Contribution %	Average dissimilarity	Contribution %
*Aedes aegypti*	4.34E-07	1.97E-06	14.97	48.81
*Aedes albopictus*	2.06E-08	9.33E-08	-	-
*Culex coronator*	5.73E-08	2.60E-07	0.7431	2.423
*Culex nigripalpus*	1.696	7.697	0.3822	1.246
*Culex quinquefasciatus*	6.16E-07	2.79E-06	1.148	3.743

### Effectiveness of adulticide spraying at different hours of the day

We leveraged a model of the mosquito population dynamics to test the effectiveness of adulticide interventions at different times of the day (S2 Fig). Specifically, we considered two scenarios representing two distinct epidemiological situations. Scenario one is during an ongoing arboviral outbreak in which the mosquitoes vector populations must be reduced quickly to curtail the epidemic. Scenario two is an effort to decrease the likelihood of an arboviral outbreak by reducing the sizes of the mosquito vector populations. In scenario two, adulticide applications may be especially important in low-resource settings or in specific situations such as before outdoor sporting events or during holidays in touristic areas when source reduction or the application of larvicides are not practical. Therefore, two different adulticide application regimes to reduce adult mosquito population sizes were evaluated; (i) adulticide applications twice a day for five consecutive days and (ii) adulticide applications once a week for two months. The adulticide treatment regimens were developed to simulate distinct epidemiological scenarios that are commonly faced by local mosquito control authorities. Input from local vector control agencies was considered to define these treatment regimes, but no official recommendation was available. For sensitivity analysis, we considered two alternative frequencies, once a month for two months and once every other week for two months. The main analysis considers the adulticide to be 50% effective; 30% and 70% are used for sensitivity analyses presented in the Supplementary Material (S3 and S4 Figs).

**Fig 2:**
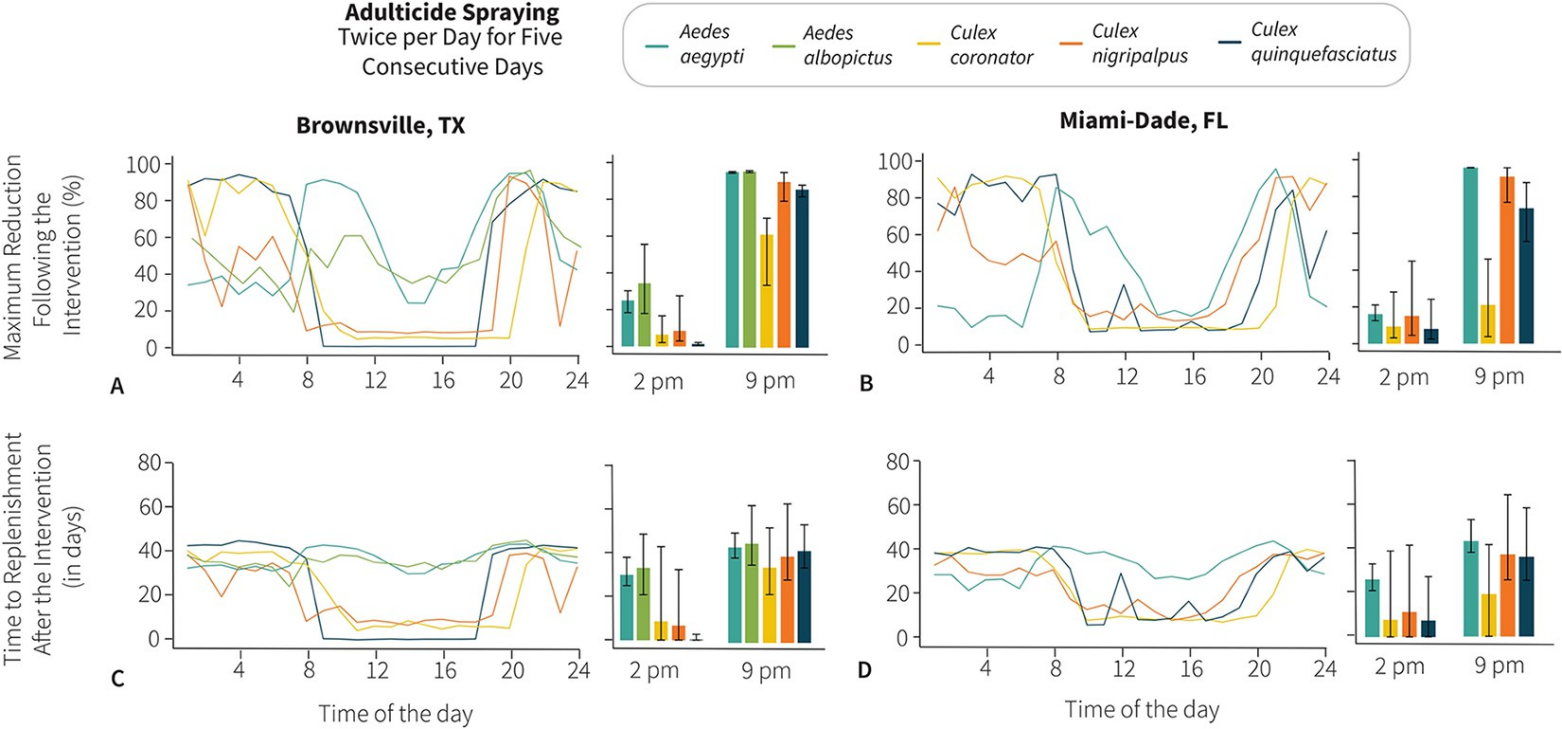
Effectiveness of adulticide application twice per day for five consecutive days by hour of application. **(A)** Maximum reduction (%) of the number of mosquitoes following adulticide application by hour of application in Brownsville, Texas. Adulticide was applied twice per day (within the same hour) for five consecutive days. The maximum reduction is obtained after the fifth day of insecticide application; after that, the mosquito population starts to recover. Lines represent mean values, which are obtained by averaging over 1,000 stochastic model realizations. Bar plots show mean and 95%CI of the same metric for adulticide application at 2 PM and 9 PM. Spraying efficacy is set at 50%. **(B)** Same as A, but for Miami-Dade County, Florida. **(C)** Number of days after the end of the adulticide application it takes for the mosquito population to recover to the pre-intervention level by hour of adulticide application. Adulticide was applied twice per day (within the same hour) for five consecutive days. Lines represent mean values, which are obtained by averaging over 1,000 stochastic model realizations. Bar plots show mean and 95%CI of the same metric for adulticide application at 2 PM and 9 PM. Intervention efficacy is set at 50%. **(D)** Same as C, but for Miami-Dade County, Florida.

The estimated effectiveness of adulticide spraying to control populations of adult vector mosquito species varied greatly according to the hour of the intervention for both daily and weekly applications. Regarding the spraying of adulticide twice per day for 5 consecutive days, even though *Ae*. *aegypti* was collected during all 24 hours of the day, it was more abundant in the morning and evening and the maximum effectiveness was obtained between 8 AM and 12 PM and 7 PM and 11 PM in Brownsville and Miami-Dade County, respectively. Our results show that adulticide interventions done during these hours of the day reach more than 80% effectiveness in reducing *Ae*. *aegypti* populations. The results for *Ae*. *albopictus* indicate that adulticide interventions in the evening (between 5 PM and 11 PM) were the most effective reaching a maximum 95.9% reduction (95% CI: 95.3 to 96.4%). Similar results were found for *Cx*. *quinquefasciatus*, *Cx*. *coronator*, and *Cx*. *nigripalpus* in which adulticide applications during the day from 9 AM to 6 PM were mostly ineffective, having a negligible impact on the abundance of mosquitoes from this species. On the other hand, adulticide applications between 6 PM and 8 AM achieved the best outcomes with a maximum of 95.7% reduction (95% CI: 95.0 to 95.9%) in the abundance of these species (Fig 2A and 2B).

Furthermore, the time of the adulticide intervention was associated with the number of days it took for a mosquito population to return to abundance levels prior to the chemical intervention. It would take 43.8 (95% CI: 38.9 to 50.4) days for the *Ae*. *aegypti* population to return to abundance levels prior to the adulticide intervention at its peak activity hours, and adulticide interventions during hours *Ae*. *aegypti* is not active lead to a less effective reduction, taking 30.2 (95% CI: 25.2 to 38.3) days to return to the abundance levels prior to the chemical intervention.

Adulticide interventions between 6 PM to 8 AM were the most effective in substantially reducing *Cx*. *coronator*, *Cx*. *nigripalpus*, and *Cx*. *quinquefasciatus* mosquitoes. As a result, it would take 42.1 (95% CI: 34.3 to 54.2) days for the *Cx*. *quinquefasciatus* population to reach the same level prior to the adulticide intervention, 34.5 (95% CI: 22.1 to 52.8) days for *Cx*. *coronator*, and 39.5 (95% CI: 28.8 to 63.7) days for *Cx*. *nigripalpus*. However, adulticide interventions were much less effective and often generated negligible results in reducing *Culex* populations if carried out between 8 AM and 6 PM, especially in Brownsville (Figs 2C, 2D and S5).

The results for adulticide application once per week for two months indicate that mosquito populations can be reduced by up to 40.7% when the intervention is carried out while mosquitoes are more active. The *Ae*. *aegypti* population from Brownsville was substantially reduced when the adulticide application was carried out between 8 AM and 12 PM and reached optimum results at 8 PM and 9 PM, whereas in Miami-Dade County adulticide applications in the morning were not as effective as in Brownsville, and the most reduction of 39.8% (95% CI: 39.6 to 40.1%) was obtained in the evening at 9 PM. The results for *Ae*. *albopictus* in Brownsville were similar to those for *Ae*. *aegypti* in Miami-Dade County. The most effective hour to control *Ae*. *albopictus* populations was between 7 PM and 10 PM, reaching a 40.7% (95% CI: 39.6 to 42.0%) reduction at 9 PM. Adulticide application at any other hour of the day resulted in a maximum of 15.9% reduction (95% CI: 9.9 to 22.8%).

**Fig 3:**
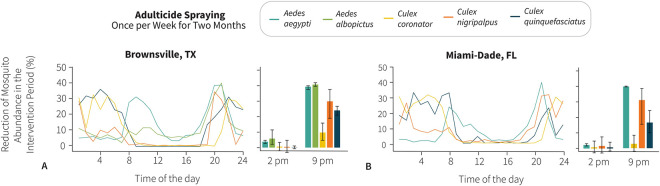
Effectiveness of adulticide application once per week for two months by hour of application. **(A)** Average reduction (%) of the number of mosquitoes following adulticide application by hour of application in Brownsville, Texas. Adulticide was applied once per week for two months. Averages are calculated over the 2-month duration of the intervention. Lines represent mean values, which are obtained by averaging over 1,000 stochastic model realizations. Bar plots show mean and 95%CI of the same metric for adulticide application at 2 PM and 9 PM. Insecticide efficacy is set at 50%. **(B)** Same as A, but for Miami-Dade County, Florida.

The results for *Cx*. *quinquefasciatus* and *Cx*. *coronator* were similar in both Brownsville and Miami-Dade County. In Brownsville, the maximum reduction was between 7 PM and 8 AM (36.7% reduction at 4 AM; 95% CI: 36.2 to 37.3%) for *Cx*. *quinquefasciatus* and 9 PM and 7 AM (33.2% reduction at 3 AM; 95% CI: 23.7 to 37.6%) for *Cx*. *coronator*. In Miami-Dade County, the maximum reduction was between 9 PM and 8 AM (31.7% at 5 AM; 95% CI: 20.2 to 38.1%) for *Cx*. *coronator* and between 9 PM and 9 AM (33.3% reduction with 95% CI: 22.6 to 37.8% at 3 AM; 31.1% reduction with 95% CI: 20.3 to 37.5% at 7 AM) for *Cx*. *quinquefasciatus*. The most effective hour of the day to control *Cx*. *nigripalpus* was found to be between 9 PM to 11 PM (29.9% reduction at 9 PM; 95% CI: 18.9 to 37.6%) and between 12 AM to 2 AM in Brownsville. In Miami-Dade County, the most effective hour of the day to control *Cx*. *nigripalpus* was between 8 PM and 2 AM (31.0% reduction with 95% CI: 15.4 to 38.5%) and at 9 PM (31.9% reduction with 95% CI: 19.4 to 39.1%) (Figs 3 and S6).

In terms of timing of adulticide application, results assuming a different frequency of the intervention and those assuming a different efficacy of the adulticide are consistent with those reported in the main analysis, albeit with lower absolute reductions (S3 and S4 Fig).

## Discussion

Scientific evidence on data on diel activity patterns of different vector mosquito species in different areas is lacking. This is because diel activity patterns are not routinely investigated during the development and implementation of mosquito control strategies. The current mosquito control strategies try to target peak activity times for adulticide applications, but rarely account for local and geographic variations in activity patterns of the target vector populations, thus, failing to reach the maximum effectiveness in controlling adult populations of the vector species. Our results showed that the mosquito community composition and abundance varied significantly throughout the day in both Brownsville, Texas, and Miami-Dade County, Florida. However, in Brownsville, epidemiologically important mosquito species did not significantly contribute to the hourly variation in the mosquito community composition, whereas in Miami-Dade County, *Ae*. *aegypti*, was the most dominant species and accounted for approximately 50% of the variation in the mosquito community composition. Even though fewer species were collected in Brownsville and *Ae*. *aegypti* was the dominant species from 8 AM to 5 PM, other epidemiologically relevant mosquito species (e.g., *Cx*. *quinquefasciatus* and *Ae*. *albopictus*) were abundantly found between dusk and dawn. On the other hand, *Ae*. *aegypti* was the most abundant species collected in Miami-Dade County at all hours of the day, and despite the higher species richness when compared to Brownsville, the bimodal diel activity pattern of *Ae*. *aegypti* was a major driver for the variation found in the mosquito community composition.

Considering the natural variations in the diel activity patterns of mosquito vector species present in Brownsville and Miami-Dade County, we modeled adulticide applications at every hour of the day. Our results indicate that the effectiveness of adulticide applications varied greatly according to the hour of the day and most effective at the peak activity time of the target mosquito vector species. Should the intervention be aimed at reducing mosquito populations as much as possible in the shortest amount of time, as would be the case when an arbovirus outbreak is ongoing, we simulated a scenario where the adulticide is sprayed twice per day for 5 consecutive days. In the case of *Ae*. *aegypti* (the primary vector of Zika, dengue, and chikungunya viruses in the study locations) in both Brownsville and Miami-Dade County, targeting the adulticide application when they are most active, 8 AM and 8 PM, resulted in the reduction of approximately 90% of the population. However, adulticide applications targeting *Ae*. *aegypti* in the early morning (between 1 AM and 7 AM) reduced only around 40% of the population. By targeting *Ae*. *aegypti* at its peak activity hour, it would take approximately 60 days for the population to reach the same abundance as prior to the adulticide intervention. However, targeting *Ae*. *aegypti* in the early morning would result in a less effective mosquito control intervention with the population reaching the same numbers as prior to the adulticide intervention after 40 days. Our results show that a simple change in the adulticide spraying from 7 AM to 8 AM would substantially impact the effectiveness of the mosquito control intervention. Spraying adulticide twice per day for 5 consecutive days yielded even more pronounced results for *Cx*. *coronator*, *Cx*. *nigripalpus*, and *Cx*. *quinquefasciatus*. Our results show that spraying adulticide between 9 AM and 6 PM achieved a negligible reduction in the *Culex* species. On the other hand, adulticide spraying at 9 PM led to a 90% reduction in the number of mosquitoes. As a result, just one hour difference in targeting *Cx*. *quinquefasciatus* can lead to a 70% reduction at 7 PM as opposed to a negligible reduction at 6 PM. Similar results were found for *Cx*. *coronator* and *Cx*. *nigripalpus*, in which spraying at 10 PM led to a 90% reduction but at 8 PM the reduction would have been negligible. By targeting the *Culex* species at their peak activity time, their populations would need approximately 50 days to rebound to the levels prior to the adulticide spraying in contrast with no reduction if the intervention would have been done just a few hours earlier. From an epidemiological standpoint, it is important to note that a 90% reduction in the vector population over a time span of 5 days (i.e., less than a generation time of Zika, dengue, and chikungunya [41–43]) would have a substantial effect on the control of an arbovirus outbreak, causing reproduction numbers of up to 10 to fall below the epidemic threshold of 1. In fact, the reproduction number (i.e., the number of secondary infections generated by an infective host through vector bites in a fully susceptible host population) for a mosquito-borne infectious disease is nearly directly proportional to the number of adult female vectors per human host. Further modelling analyses simulating the pathogen transmission dynamics would be warranted to quantify the non-linear effect of adulticide application on key epidemiological metrics such as the size and duration of an epidemic outbreak, and disease burden. It is also important to stress that incorporating mosquito behavior patterns other than host-seeking (e.g., sugar seeking) could provide more reliable estimations of the effect of adulticide application.

When interventions are aimed at keeping mosquito vector populations to acceptable levels, for instance, to decrease the likelihood of an arbovirus outbreak, we simulated spraying adulticide in more sparse intervals for two months (e.g., once per week, once every other week, and once per month). Also, in this case, the effectiveness of the intervention was positively associated with the peak activity time of each target mosquito vector species. For instance, for *Ae*. *aegypti*, the estimated reduction ranged from 20.1% (95% CI: 19.7 to 20.6%) and 20.1% (95% CI: 19.5 to 20.4%) when spraying is performed at 9 PM in both Miami-Dade County and Brownsville to 4.3% (95% CI: 3.1 to 5.3%) and 2.8% (95% CI: 1.9 to 3.8%) when spraying is performed at 2 PM.

Our modeling analysis considers the worst-case scenario in which the temperature is kept constant at 25 degrees Celsius–an optimal temperature for mosquito development and proliferation [32]. As such, our estimates can be considered as a lower bound for the effectiveness of the intervention and are not able to capture the natural seasonal variation in mosquito relative abundance [31,44] and diel activity patterns [17]. Further analyses are needed to provide operational guidance for vector control operations accounting for seasonal variations. A limitation of our analysis is due to the unavailability of the development and mortality ratios for *Cx*. *coronator* and *Cx*. *nigripalpus* in the scientific literature, we have used the parameters that were available for *Cx*. *quinquefasciatus*. The mosquito species of the *Culex* genus are organized into 26 subgenera, and both *Cx*. *coronator* and *Cx*. *nigripalpus* share the same subgeneric classification as *Cx*. *quinquefasciatus*; they all belong to the subgenus *Culex* [45]. Therefore, even though these are different species they share similar biological traits and the parameters of *Cx*. *quinquefasciatus* are reasonable approximations for *Cx*. *coronator* and *Cx*. *nigripalpus* (S5 Table). Another limitation is that the model considers a single mosquito population at the time, thus ignoring possible competition between species. However, we do not expect the competition for resources to be an important driver for the proliferation of the species selected for this study as they exploit different resources in the urban environment and have distinct population dynamics [46,47]. Finally, the model summarizes into a single parameter all of the complexities determining the carrying capacity of the environment, such as larval habitat availability, possible drying out of larval habitats, immature mosquitoes could be washed out from larval habitats, larval habitat condition (e.g., tires can insulate immature mosquitoes from the elements and have low species richness, natural larval habitats may have predators as well as other species competing for resources), levels of organic material in larval habitats may follow seasonal trends. In sum, there are multiple factors determining the carrying capacity of the environment. This is captured in our modeling work by considering an overall carrying capacity that is included in the equation describing the dynamics of eggs. Therefore, our model neither has the resolution nor the aim of investigating factors determining the carrying capacity of the environment.

Adulticide spraying during the day would potentially increase the risk of exposure of the general public to adulticides and should be considered in the development of mosquito control strategies [48]. Furthermore, different propellant mechanisms (e.g., Buffalo Turbine, Grizzly ULV Sprayer, airplane, etc.) may have different levels of effectiveness due to intrinsic limitations in their operation mechanisms, droplet size, as well as due to different weather conditions (e.g., air temperature and wind speed), presence of vegetation, and other factors present in the target area.

Despite being one of the most important aspects for the development of effective mosquito control strategies based on adulticide spraying and assessing the exposure risk to infective bites, the only recent studies focusing on the diel activity patterns of *Ae*. *aegypti* in the contiguous United States were done in St. Augustine, Florida, in 2017 by Smith et. al. [49] and in 2020 in Miami-Dade County, Florida, and Brownsville, Texas, by Mutebi et al. [17]. Both studies show a clear bimodal pattern of peak activity of *Ae*. *aegypti* in the morning and evening.

Adulticides are commonly used in the United States during emergency situations to control and prevent arbovirus outbreaks. For instance, they were widely used during the Zika virus outbreak in Miami-Dade County, Florida [3,50,51]. Our results have great implications to improve the effectiveness of mosquito control strategies. Indeed, diel activity patterns of vector mosquito species should be considered when developing future mosquito control strategies.

In case of an arbovirus outbreak, in addition to adulticide application, it is common practice, as well as recommended by CDC guidelines for surveillance, prevention, and control of West Nile virus [52,53], to establish a perimeter and remove potential aquatic habitats, spray larvicide and adulticide at the point source location of the infection and patient’s home address in response to imported and locally transmitted arboviral infections [46,54]. The use of adulticide disregarding the diel activity of the target mosquito species can lead not only to ineffective control interventions but, more importantly, the indiscriminate use of adulticide, even during emergency situations, can have deleterious results by increasing the levels of adulticide resistance of the target mosquito population which may lead to decreased effectiveness of adulticide(s) [15,55,56]. Targeting adult mosquito vector species with adulticide spraying-based control strategies during their peak activity will not only result in more effective control strategies but will also result in the need to spray adulticides fewer times, potentially reducing the development of adulticide resistance by the target mosquito species and the exposure of non-target species.

## Conclusion

This study serves as a cornerstone for future studies to improve the effectiveness of mosquito control strategies that employ adulticide applications. Our results point to a well-defined diel activity pattern in the peak activity of mosquito species reaching maximum relative abundance at specific times in both Brownsville, Texas, and Miami-Dade County, Florida. Furthermore, the effectiveness of simulated adulticide applications varied greatly according to the hour of the day with 9 PM being the most effective time of the day to control populations of all vector mosquito species in Miami-Dade County and Brownsville. Our results indicate that the timing of adulticide spraying interventions should be carefully considered by local authorities based on the ecology of mosquito species in the focus area.

## Disclaimer

The information provided, and views expressed in this publication do not necessarily reflect the official position of the Centers for Disease Control and Prevention (CDC).

## Supporting information

S1 Table Female mosquitoes captured by BG-Sentinel 2 traps in Brownsville, Texas. (PDF)

S2 Table Female mosquitoes captured by BG-Sentinel 2 traps in Miami-Dade, Florida. (PDF)

S3 Table SIMPER (Similarity Percentage) analysis of which species contributed the most to the observed differences in Brownsville, Texas. (PDF)

S4 Table SIMPER (Similarity Percentage) analysis of which species contributed the most to the observed differences in Miami-Dade, Florida. (PDF)

S5 Table Mosquito species development and mortality ratios. (PDF)

S1 Fig Diel activity patterns of mosquito populations. **(A)** Total number of collected female adult mosquito specimens collected over the entire study period by hour of collection in Brownsville, Texas. The Dominance Index is shown in black (mean and 95% CI). **(B)** Same as A, but for Miami-Dade County.
(TIF)

S2 Fig Simulated temporal dynamics considering adulticide application. **(A)** Simulate number of adult females of *Ae*. *aegypti* when considering adulticide application twice a day at 9 PM for five consecutive days in Brownsville, Texas. Insecticide efficacy is set at 50%. **(B)** Same as A, but adulticide is applied once per week for two months.
(TIF)

S3 Fig Effectiveness of adulticide application for different frequencies of application. **(A)** Average reduction (%) of the number of mosquitoes following adulticide application by hour of application in Brownsville, Texas. Averages are calculated over the 2-month duration of the intervention. Lines represent mean values and shaded areas represent 95%CI; results are obtained by analyzing 1,000 stochastic model realizations. Insecticide efficacy is set at 50%. **(B)** Same as A, but for Miami-Dade County, Florida.
(TIFF)

S4 Fig Effectiveness of adulticide application once per week for two months for different insecticide efficacies. **(A)** Average reduction (%) of the number of mosquitoes following adulticide application by hour of application in Brownsville, Texas. Adulticide was applied once per week for two months. Averages are calculated over the 2-month duration of the intervention. Lines represent mean values and shaded areas represent 95%CI; results are obtained by analyzing 1,000 stochastic model realizations. **(B)** Same as A, but for Miami-Dade County, Florida.
(TIF)

S5 Fig Effectiveness of adulticide application twice per day for five consecutive days by hour of application. **(A)** Number of days after the end of the adulticide application it takes for the mosquito population to recover to the pre-intervention level by hour of adulticide application. Adulticide was applied twice per day (within the same hour) for five consecutive days. Lines represent mean values and shaded areas represent 95%CI; results are obtained by analyzing 1,000 stochastic model realizations. Insecticide efficacy is set at 50%. **(B)** Same as A, but for Miami-Dade County, Florida.
(TIF)

S6 Fig Effectiveness of adulticide application once per week for two months by hour of application. **(A)** Average reduction (%) of the number of mosquitoes following adulticide application by hour of application in Brownsville, Texas. Adulticide was applied once per week for two months. Averages are calculated over the 2-month duration of the intervention. Lines represent mean values and shaded areas represent 95%CI; results are obtained by analyzing 1,000 stochastic model realizations. Insecticide efficacy is set at 50%. **(B)** Same as A, but for Miami-Dade County, Florida.
(TIF)
