# Supplementary material for: Diel activity patterns of vector mosquito species in the urban environment: Implications for vector control strategies
Source: PLoS Negl Trop Dis. 2023 Jan 26;17(1):e0011074. doi: 10.1371/journal.pntd.0011074 (PMC9879453; doi:10.1371/journal.pntd.0011074)
Supplement: S1 Table — (PDF) [file pntd.0011074.s001.pdf]

Supplementary Table 1. Female mosquitoes captured by BG-Sentinel 2 traps in Brownsville, Texas.

Species	12 AM	1 AM	2 AM	3 AM	4 AM	5 AM	6 AM	7 AM	8 AM	9 AM	10 AM	11 AM	12 PM	1 PM	2 PM	3 PM	4 PM	5 PM	6 PM	7 PM	8 PM	9 PM	10 PM	11 PM
Aedes aegypti	96	103	116	82	104	78	106	582	673	591	473	252	129	66	66	130	134	258	488	870	870	476	151	130
Aedes albopictus	17	14	11	8	11	8	4	15	10	18	18	11	9	8	9	8	11	13	33	57	73	25	18	15
Aedes vexans	78	82	94	58	74	78	104	39	2	2	3		1	1		3	12	1	8	22	76	174	128	105
Aedes infirmatus	17	14	23	10	21	12	28	11	2	2	2					1	7	1		34	70	91	26	23
Aedes sollicitans	8	4		5	7	6	9				1								2		1	6	6	3
Aedes taeniorhynchus	25	28	16	27	13	31	37	25	4		3	1								10	83	43	45	29
Culex quinquefasciatus	340	426	403	491	432	289	269	110											164	226	297	412	320	294
Culex nigripalpus	19	5	2	6	5	7	4		1	1										24	20	12	1	6
Culex coronator	39	13	42	27	39	33	16	10	3	1											11	37	35	29
Anopheles pseudopunctipennis	2	2	2	6	1	3	1													2			3	5
Psorophora cyanescens					1	1							1			2	5			7	8	2	1	
Psorophora columbiae	1	4	3	3	1	6	2	2													3	3	2	3
Aedes thelcter			1																		8	1	2	
Culex erraticus				1	4	3																	1	
Toxorhynchites rutilus															1					1	2			
Psorophora ciliata						1																		
Aedes zoosophus																							1	
