# Supplementary material for: Diel activity patterns of vector mosquito species in the urban environment: Implications for vector control strategies
Source: PLoS Negl Trop Dis. 2023 Jan 26;17(1):e0011074. doi: 10.1371/journal.pntd.0011074 (PMC9879453; doi:10.1371/journal.pntd.0011074)
Supplement: S2 Table — (PDF) [file pntd.0011074.s002.pdf]

Supplementary Table 2. Female mosquitoes captured by BG-Sentinel 2 traps in Miami-Dade, Florida.

Species	12 AM	1 AM	2 AM	3 AM	4 AM	5 AM	6 AM	7 AM	8 AM	9 AM	10 AM	11 AM	12 PM	1 PM	2 PM	3 PM	4 PM	5 PM	6 PM	7 PM	8 PM	9 PM	10 PM	11 PM
Aedes aegypti	56	53	24	41	42	24	124	504	401	221	254	160	107	43	50	42	55	132	236	478	931	337	74	55
Aedes albopictus																		1						
Aedes taeniorhynchus	181	188	162	139	162	155	129	69	62	33	42	61	28	26	25	20	21	26	32	28	138	382	254	217
Aedes tortilis	17	16	21	17	6	18	25	43	34	13	27	43	13	11	14	9	12	13	13	24	111	73	30	26
Aedes triseriatus								1									1				1	1	2	
Anopheles atropos	1																							
Anopheles crucians	2	2	2	1					1													1	2	1
Anopheles quadrimaculatus	2		4	2	1		1															1		7
Culex biscaynensis								10	2					1							1			
Culex coronator	22	14	18	20	22	21	16	5	2												2	13	21	18
Culex erraticus	1		1			1		3														1	1	
Culex nigripalpus	5	11	4	3	3	4	3	4	1		1		1					1	3	4	13	13	6	11
Culex quinquefasciatus	15	12	30	23	23	16	27	29	5			4				1			1	4	13	20	4	10
Deinocerites cancer	1	1		3	1			3					1									2	3	1
Psorophora columbiae							2								4	2					3	30	3	1
Wyeomyia mitchellii											1	1	1	1	1		2	2	1	1				
Wyeomyia vanduzeei		1		1	1	7	36	157	169	158	199	187	189	136	140	149	167	183	259	294	195	7	2	2
Aedes infirmatus					1																			1
