# Supplementary material for: Diel activity patterns of vector mosquito species in the urban environment: Implications for vector control strategies
Source: PLoS Negl Trop Dis. 2023 Jan 26;17(1):e0011074. doi: 10.1371/journal.pntd.0011074 (PMC9879453; doi:10.1371/journal.pntd.0011074)
Supplement: S3 Table — (PDF) [file pntd.0011074.s003.pdf]

Supplementary Table 3. SIMPER (Similarity Percentage) analysis of which species contributed the most to the observed differences in Brownsville, Texas.

Species	Average dissimilarity	Contribution %	Cumulative contribution %	Mean 1	Mean 2
Psorophora cyanescens	5.434	24.65	24.65	2.15E+09	1.07E+09
Culex erraticus	4.973	22.56	47.22	1.61E+09	1.07E+09
Aedes sollicitans	3.12	14.16	61.37	5.37E+08	5.37E+08
Psorophora ciliata	2.865	13	74.37	2.15E+09	1.61E+09
Aedes thelcter	2.257	10.24	84.61	1.61E+09	2.15E+09
Culex nigripalpus	1.696	7.697	92.3	8	5.37E+08
Anopheles pseudopunctipennis	1.696	7.697	100	3	5.37E+08
Culex quinquefasciatus	6.16E-07	2.79E-06	100	415	275
Aedes aegypti	4.34E-07	1.97E-06	100	99.3	218
Aedes vexans	8.50E-08	3.86E-07	100	78	73.8
Culex coronator	5.73E-08	2.60E-07	100	30.3	24.5
Aedes taeniorhynchus	3.81E-08	1.73E-07	100	24	26.5
Aedes infirmatus	3.02E-08	1.37E-07	100	16	18
Aedes albopictus	2.06E-08	9.33E-08	100	12.5	9.5
Psorophora colombiae	8.65E-09	3.92E-08	100	2.75	2.75
Toxorhynchites rutilus			100	2.15E+09	2.15E+09
Aedes zoosophus			100	2.15E+09	2.15E+09
