# Supplementary material for: Diel activity patterns of vector mosquito species in the urban environment: Implications for vector control strategies
Source: PLoS Negl Trop Dis. 2023 Jan 26;17(1):e0011074. doi: 10.1371/journal.pntd.0011074 (PMC9879453; doi:10.1371/journal.pntd.0011074)
Supplement: S4 Table — (PDF) [file pntd.0011074.s004.pdf]

Supplementary Table 4. SIMPER (Similarity Percentage) analysis of which species contributed the most to the observed differences in Miami-Dade, Florida.

Species	Average dissimilarity	Contribution %	Cumulative contribution %	Mean 1	Mean 2
Aedes aegypti	14.97	48.81	48.81	43.5	174
Aedes taeniorhynchus	5.436	17.72	66.53	168	129
Wyeomyia vanduzeei	5.251	17.12	83.65	.5	50.3
Aedes tortilis	1.48	4.826	88.48	17.8	23
Culex quinquefasciatus	1.148	3.743	92.22	20	23.8
Culex coronator	.7431	2.423	94.65	18.5	16
Culex nigripalpus	.3822	1.246	95.89	5.75	3.5
Anopheles quadrimaculatus	.2711	.8839	96.78	2	.5
Anopheles crucians	.2702	.8811	97.66	1.75	
Culex biscaynensis	.2259	.7365	98.39		2.5
Deinocerites cancer	.1856	.6053	99	1.25	1
Culex erraticus	.1228	.4005	99.4	.5	1
Psorophora columbiae	.07794	.2541	99.65		.5
Aedes infirmatus	.04627	.1509	99.8		.25
Anopheles atropos	.03736	.1218	99.93	.25	
Aedes triseriatus	.02259	.07365	100		.25
Wyeomyia mitchellii			100		
Psorophora columbiae			100		
Aedes albopictus			100		
