# Supplementary material for: Diel activity patterns of vector mosquito species in the urban environment: Implications for vector control strategies
Source: PLoS Negl Trop Dis. 2023 Jan 26;17(1):e0011074. doi: 10.1371/journal.pntd.0011074 (PMC9879453; doi:10.1371/journal.pntd.0011074)
Supplement: S5 Table — (PDF) [file pntd.0011074.s005.pdf]

Supplementary Table 5. Mosquito species development and mortality ratios.

Parameters	Description	*Aedes aegypti*	*Aedes albopictus*	*Culex coronator*	*Culex nigripalpus*	*Culex quinquefasciatus*
d_E	Development rate from egg to larva (day^{-1})	0.31 [33]	0.16 [30]	0.57 [35]	0.57 [35]	0.57 [35]
d_L	Development rate from larva to pupa (day^{-1})	0.13 [32]	0.12 [30]	0.13 [32]	0.13 [32]	0.13 [32]
d_P	Development rate from pupa to adult (day^{-1})	0.65 [32]	0.48 [30]	0.55 [32]	0.55 [32]	0.55 [32]
d_A	Duration of Gonotrophic cycle (day^{-1})	0.2 [34]	0.20 [30]	0.25 [36]	0.25 [36]	0.25 [36]
m_E	Mortality rate from egg to larva (day^{-1})	0.32 [33]	0.16 [30]	0.17 [37, 38]	0.17 [37, 38]	0.17 [37, 38]
m_L	Mortality rate from larva to pupa (day^{-1})	0.08 [39]	0.02 [30]	0.04 [37, 38]	0.04 [37, 38]	0.04 [37, 38]
m_P	Mortality rate from pupa to adults (day^{-1})	0.06 [39]	0.02 [30]	0.01 [37, 38]	0.01 [37, 38]	0.01 [37, 38]
m_A	Mortality rate of adults (day^{-1})	0.03 [40]	0.03 [30]	0.03 [37, 38]	0.03 [37, 38]	0.03 [37, 38]
n_E	Number of eggs per oviposition	100 [33]	100 [30]	175 [37, 38]	175 [37, 38]	175 [37, 38]
