# Supplementary figures and images for: Diel activity patterns of vector mosquito species in the urban environment: Implications for vector control strategies
Source: PLoS Negl Trop Dis. 2023 Jan 26;17(1):e0011074. doi: 10.1371/journal.pntd.0011074 (PMC9879453; doi:10.1371/journal.pntd.0011074)

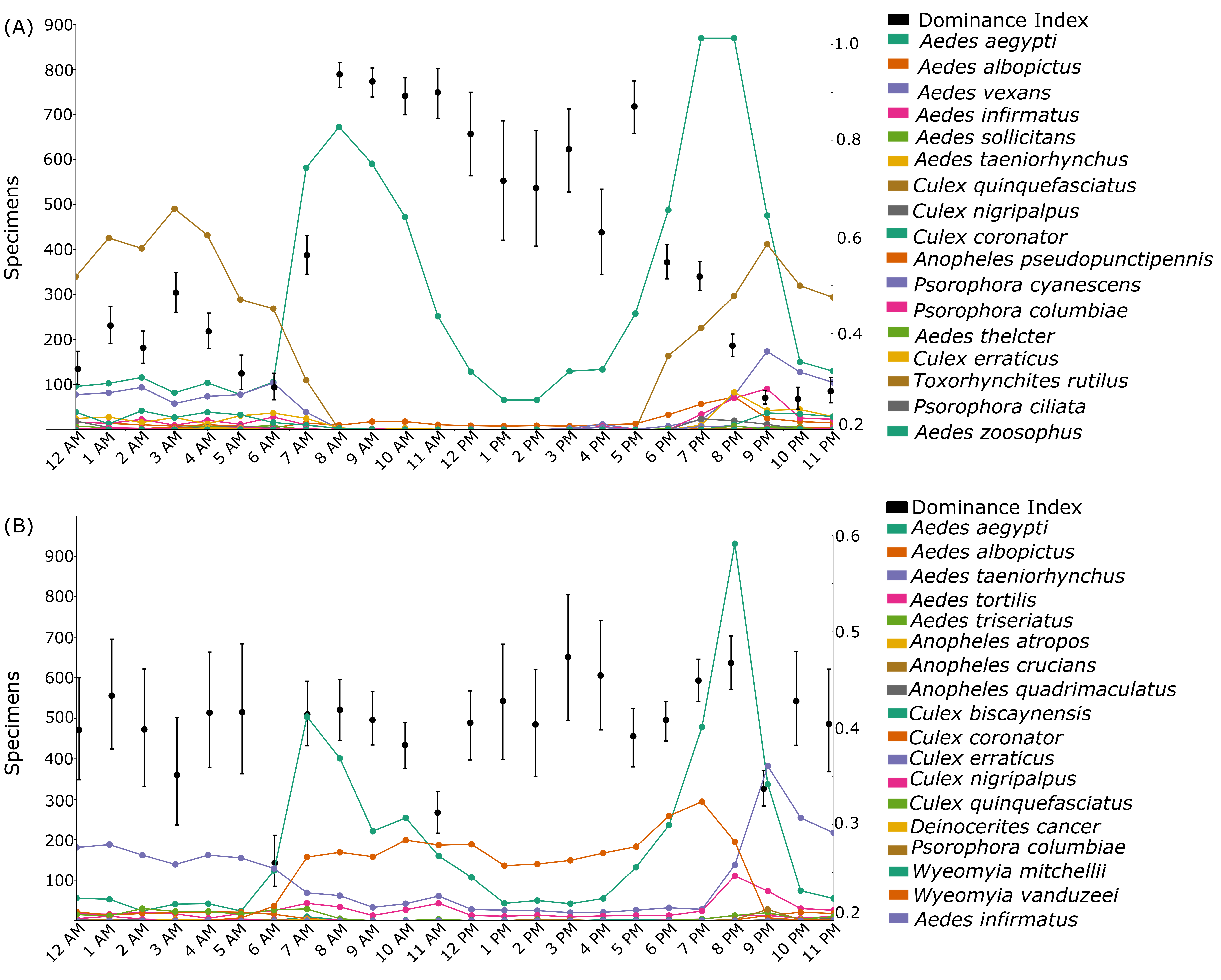

Supplement: S1 Fig — (A) Total number of collected female adult mosquito specimens collected over the entire study period by hour of collection in Brownsville, Texas. The Dominance Index is shown in black (mean and 95% CI). (B) Same as A, but for Miami-Dade County. (TIF) [file pntd.0011074.s006.tif]

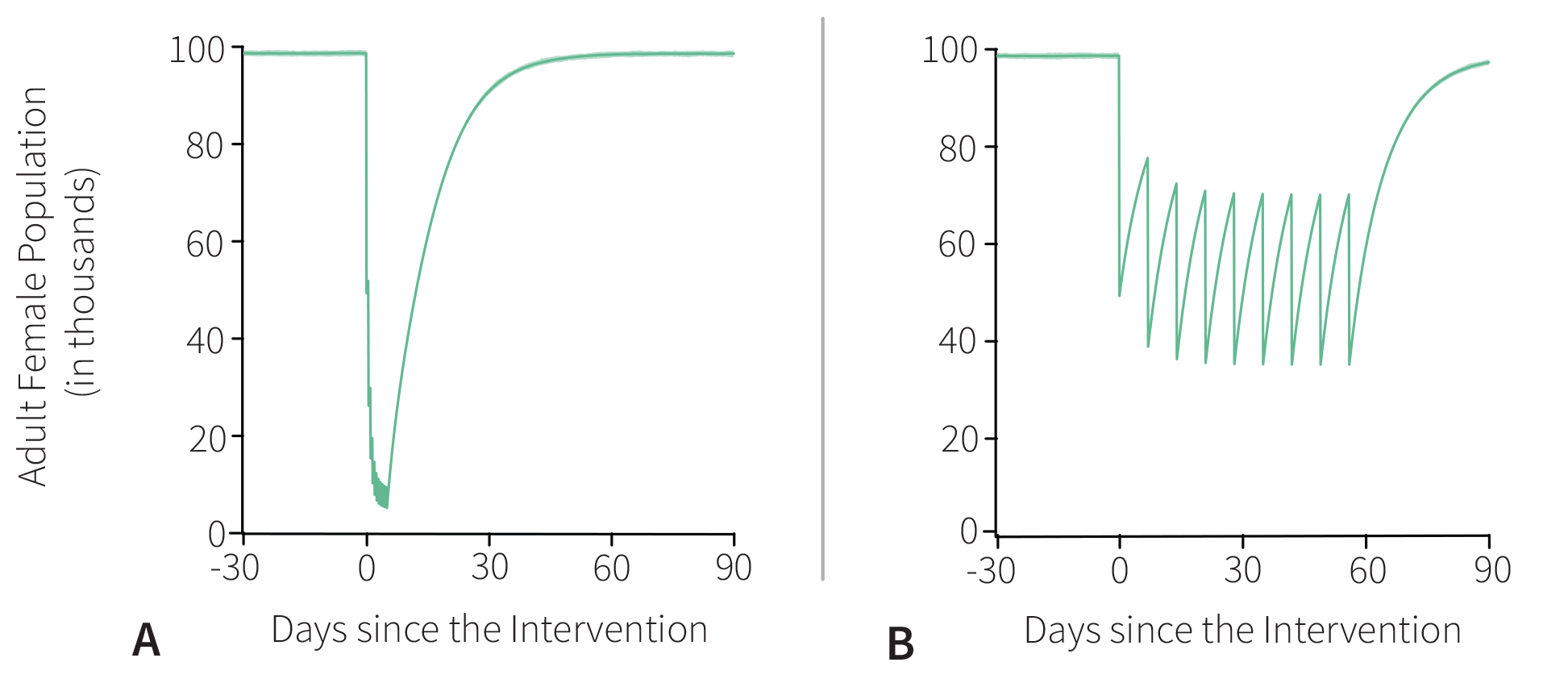

Supplement: S2 Fig — (A) Simulate number of adult females of Ae. aegypti when considering adulticide application twice a day at 9 PM for five consecutive days in Brownsville, Texas. Insecticide efficacy is set at 50%. (B) Same as A, but adulticide is applied once per week for two months. (TIF) [file pntd.0011074.s007.tif]

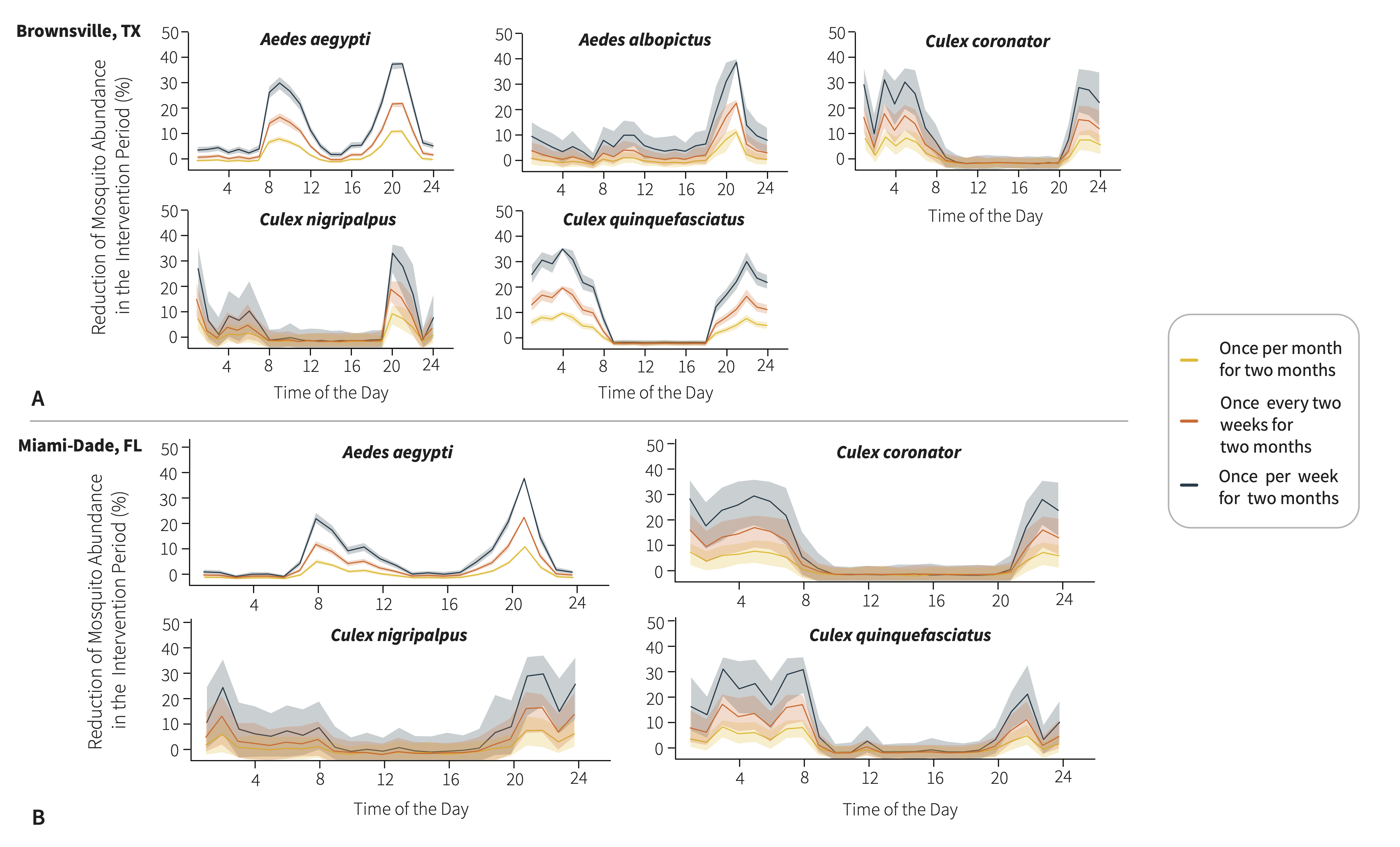

Supplement: S3 Fig — (A) Average reduction (%) of the number of mosquitoes following adulticide application by hour of application in Brownsville, Texas. Averages are calculated over the 2-month duration of the intervention. Lines represent mean values and shaded areas represent 95%CI; results are obtained by analyzing 1,000 stochastic model realizations. Insecticide efficacy is set at 50%. (B) Same as A, but for Miami-Dade County, Florida. (TIFF) [file pntd.0011074.s008.tiff]

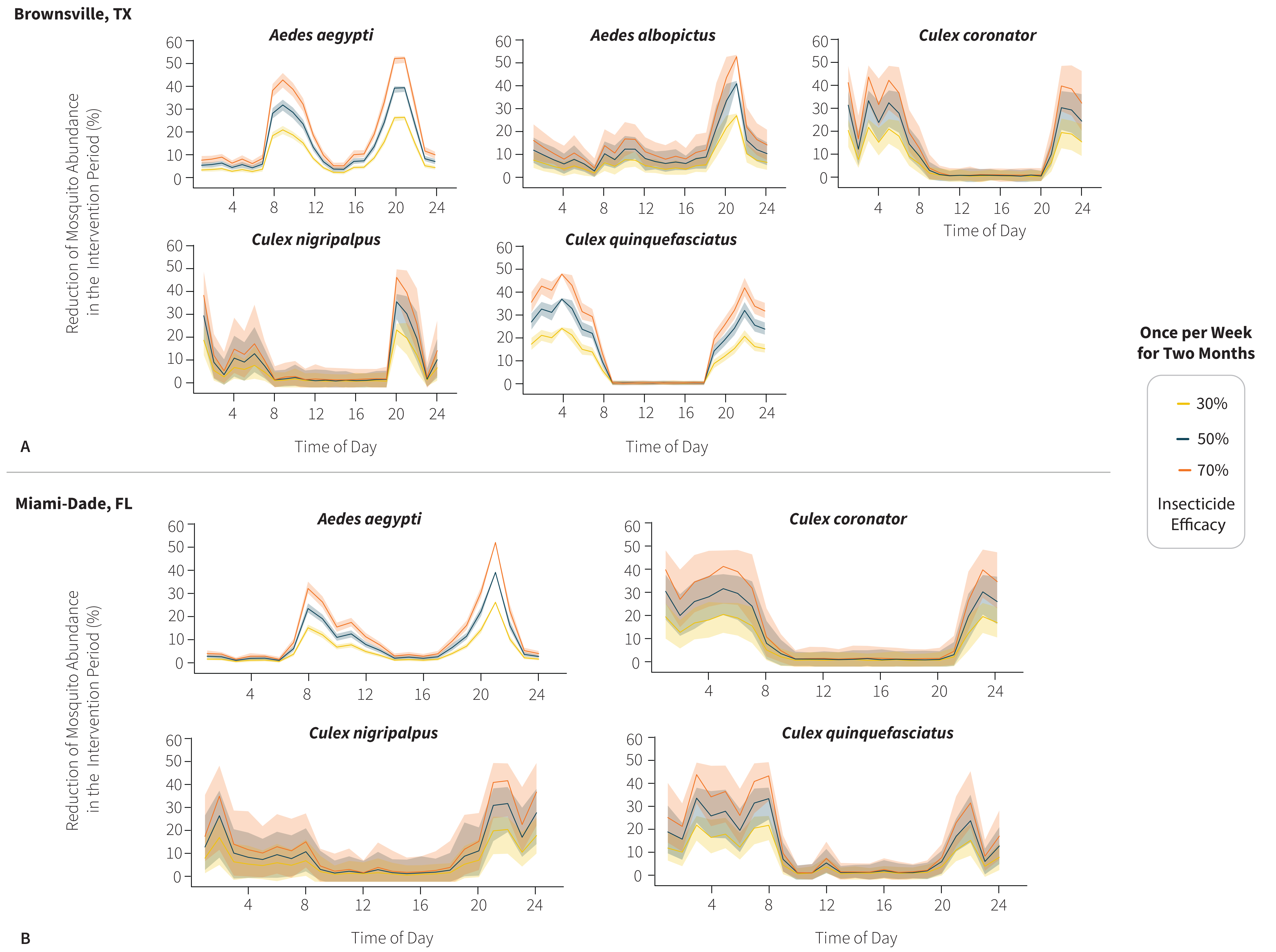

Supplement: S4 Fig — (A) Average reduction (%) of the number of mosquitoes following adulticide application by hour of application in Brownsville, Texas. Adulticide was applied once per week for two months. Averages are calculated over the 2-month duration of the intervention. Lines represent mean values and shaded areas represent 95%CI; results are obtained by analyzing 1,000 stochastic model realizations. (B) Same as A, but for Miami-Dade County, Florida. (TIF) [file pntd.0011074.s009.tif]

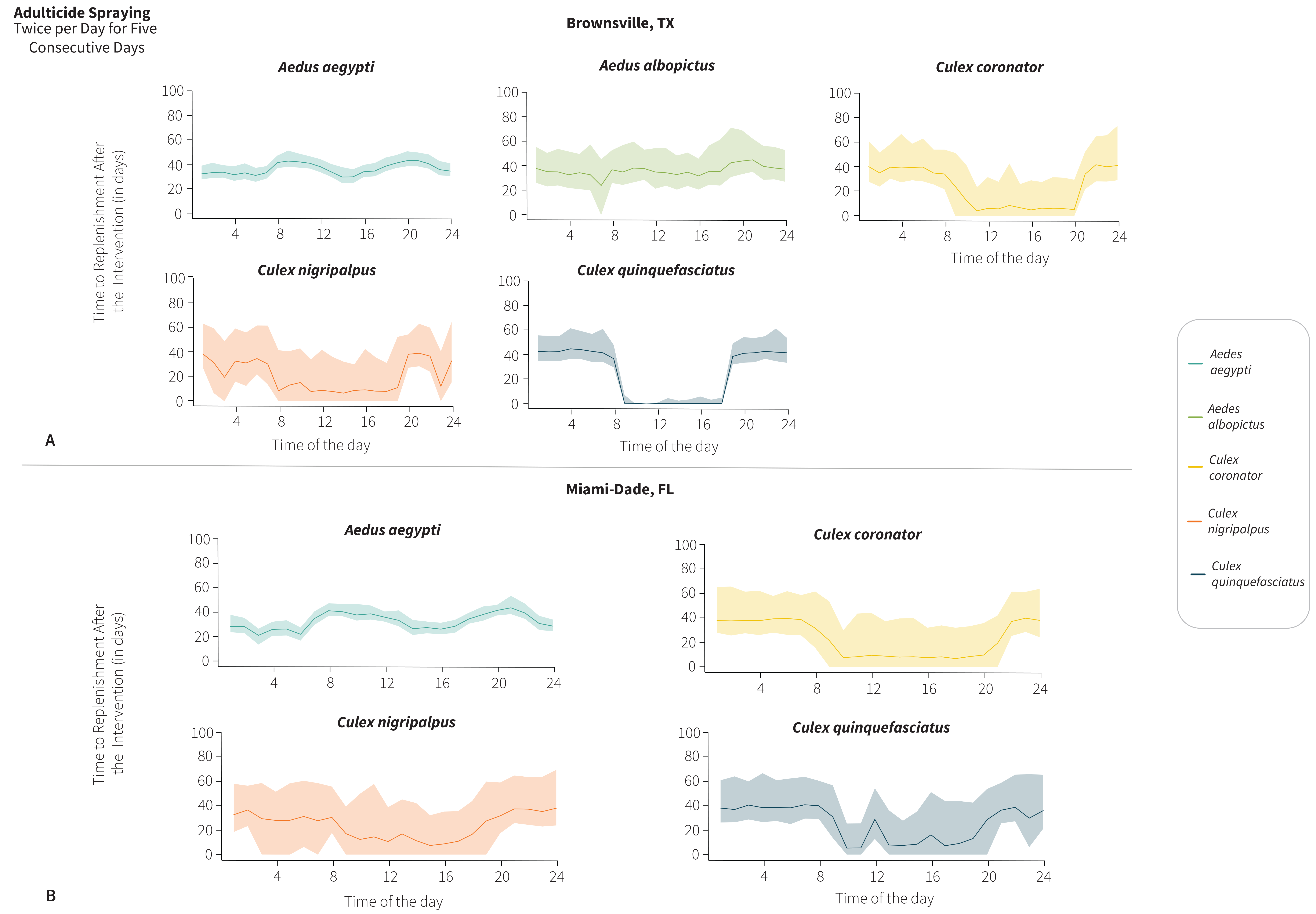

Supplement: S5 Fig — (A) Number of days after the end of the adulticide application it takes for the mosquito population to recover to the pre-intervention level by hour of adulticide application. Adulticide was applied twice per day (within the same hour) for five consecutive days. Lines represent mean values and shaded areas represent 95%CI; results are obtained by analyzing 1,000 stochastic model realizations. Insecticide efficacy is set at 50%. (B) Same as A, but for Miami-Dade County, Florida. (TIF) [file pntd.0011074.s010.tif]

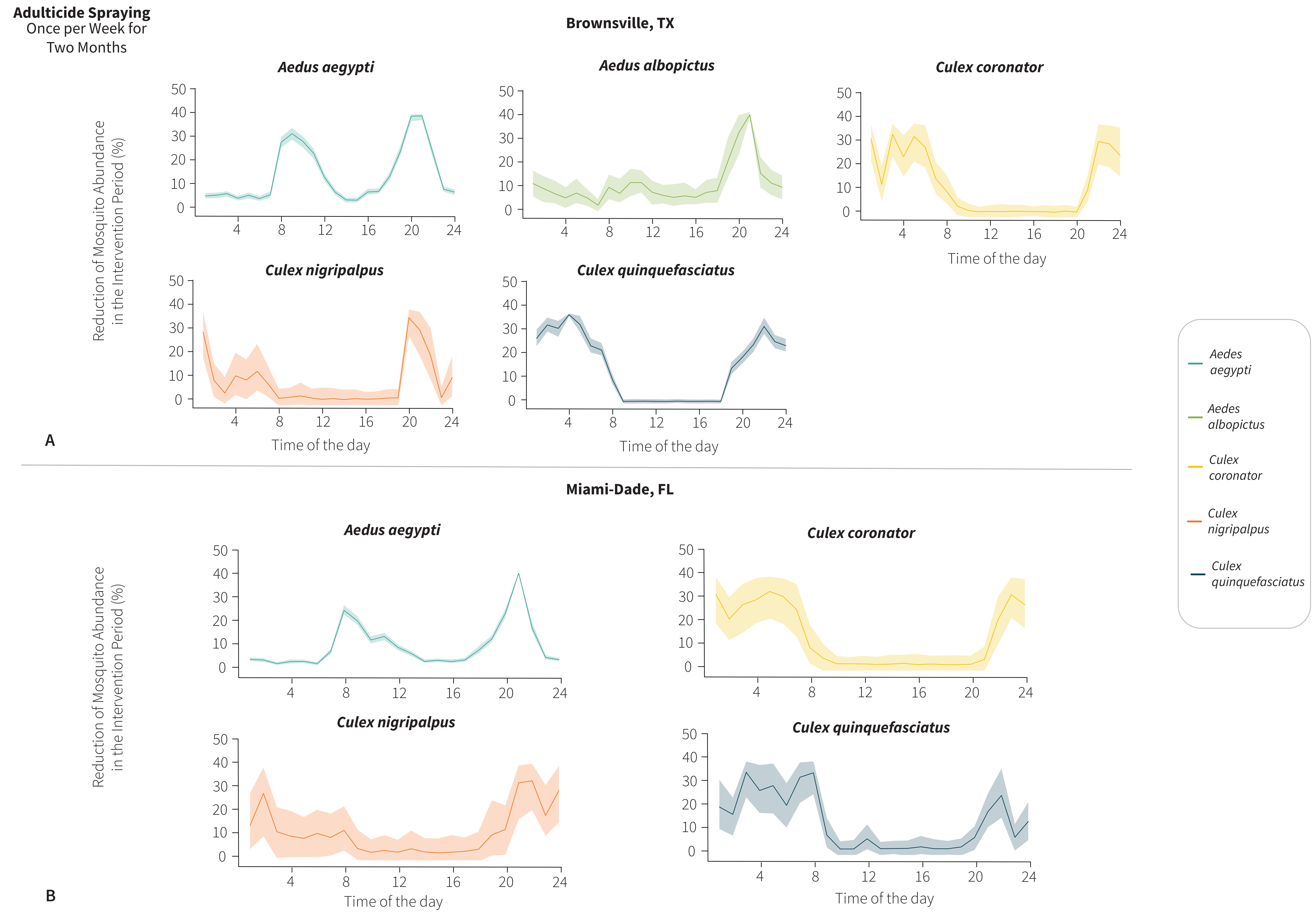

Supplement: S6 Fig — (A) Average reduction (%) of the number of mosquitoes following adulticide application by hour of application in Brownsville, Texas. Adulticide was applied once per week for two months. Averages are calculated over the 2-month duration of the intervention. Lines represent mean values and shaded areas represent 95%CI; results are obtained by analyzing 1,000 stochastic model realizations. Insecticide efficacy is set at 50%. (B) Same as A, but for Miami-Dade County, Florida. (TIF) [file pntd.0011074.s011.tif]
